# Chronic Conditions Among Adults Aged 18─34 Years — United States, 2019

**DOI:** 10.15585/mmwr.mm7130a3

**Published:** 2022-07-29

**Authors:** Kathleen B. Watson, Susan A. Carlson, Fleetwood Loustalot, Machell Town, Paul I. Eke, Craig W. Thomas, Kurt J. Greenlund

**Affiliations:** ^1^Division of Population Health, National Center for Chronic Disease Prevention and Health Promotion, CDC; ^2^Division of Heart Disease and Stroke Prevention, National Center for Chronic Disease Prevention and Health Promotion, CDC.

Chronic conditions are common, costly, and major causes of death and disability.* Addressing chronic conditions and their determinants in young adulthood can help slow disease progression and improve well-being across the life course (*1*); however, recent prevalence estimates examining chronic conditions in young adults overall and by subgroup have not been reported. CDC analyzed data from the Behavioral Risk Factor Surveillance System (BRFSS) to measure prevalence of 11 chronic conditions among adults aged 18–34 years overall and by selected characteristics, and to measure prevalence of health-related risk behaviors by chronic condition status. In 2019, more than one half (53.8%) of adults aged 18–34 years reported having at least one chronic condition, and nearly one quarter (22.3%) reported having more than one chronic condition. The most prevalent conditions were obesity (25.5%), depression (21.3%), and high blood pressure (10.7%). Differences in the prevalence of having a chronic condition were most noticeable between young adults with a disability (75.8%) and without a disability (48.3%) and those who were unemployed (62.3%) and students (45.8%). Adults aged 18–34 years with a chronic condition were more likely than those without one to report binge drinking, smoking, or physical inactivity. Coordinated efforts by public and private sectors might help raise awareness of chronic conditions among young adults and help improve the availability of evidence-based interventions, policies, and programs that are effective in preventing, treating, and managing chronic conditions among young adults (*1*).

BRFSS is an annual state-based, random-digit–dialed telephone survey of noninstitutionalized U.S. adults aged ≥18 years.^†^ In 2019, BRFSS included data from 67,104 respondents aged 18–34 years; New Jersey did not collect sufficient data to meet the minimum requirement for inclusion in the public-use data set. The median response rate for the remaining 49 states and the District of Columbia was 49.4% (range = 37.3% for New York to 73.1% for South Dakota).^§^ Having a chronic condition was defined as responding “yes” to having ever been told by a doctor or other health professional that the respondent had any of the following: a depressive disorder (depression); arthritis; a heart attack, angina, coronary heart disease, or stroke (heart disease/stroke); chronic obstructive pulmonary disease; skin or other types of cancer (cancer); kidney disease; diabetes; high cholesterol; high blood pressure; or current asthma. The five conditions with the lowest prevalence were combined into a single variable called “other.” Obesity (body mass index ≥30.0 kg/m^2^) was based on self-reported height and weight. Health-related risk behaviors included self-reported binge drinking, current smoking, and physical inactivity.^¶^

Prevalence of any condition and of each specific condition was estimated overall and by selected sociodemographic, location, and health-related characteristics, including self-rated health and access to health care. Prevalence of each health-related risk behavior was estimated by chronic condition status. Paired t-tests were conducted to identify subgroup differences among all pairs except those including other race and ethnicity and other employment status. Although all comparisons reported are statistically significant (Bonferroni-corrected p-value <0.05), only sociodemographic and location comparisons where the prevalence ratio is >1.3 will be discussed. Multiple imputation techniques were used to account for missing data.** SAS (version 9.4; SAS Institute) and SUDAAN (version 11.0; RTI International) were used to account for survey weights and the complex sampling design. This activity was reviewed by CDC and was conducted consistent with applicable federal law and CDC policy.^††^

Overall, 53.8% (39.8 million) of adults aged 18–34 years had at least one of the 11 conditions, and 22.3% had more than one condition (Figure 1). The most frequently reported conditions were obesity (25.5%), depression (21.3%), and high blood pressure (10.7%), and more than one half (ranging from 53.9% among adults with obesity to among 86.0% of adults with diabetes) of those with a specific condition had at least one other condition. For example, although 25.5% of young adults had obesity, 13.7% of young adults had obesity and at least one other condition. Having any chronic condition was significantly associated with all selected characteristics. Differences in the prevalence of having any condition by sociodemographic and location characteristics were most noticeable between young adults with a disability (75.8%) and those without a disability (48.3%) and those who were unemployed (62.3%) and a student (45.8%) (Table).

**FIGURE 1:**
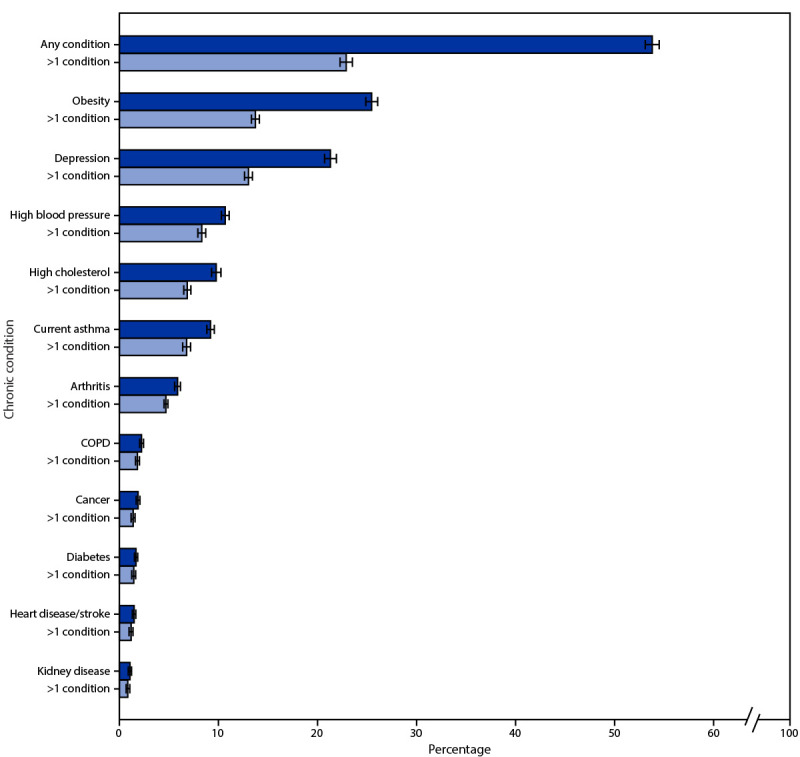
Percentage* of chronic conditions^†^ among adults aged 18–34 years — Behavioral Risk Factor Surveillance System, United States, 2019 **Abbreviation:** COPD = chronic obstructive pulmonary disease. * 95% CIs indicated by error bars. ^†^ Behavioral Risk Factor Surveillance System respondents were classified as having a chronic condition if they had a body mass index >30.0 kg/m^2^ or if they had ever been told by a doctor, nurse, or other health professional they had any of the following conditions: depression, arthritis, heart disease/stroke, COPD, cancer, kidney disease, diabetes, high cholesterol, high blood pressure, or currently have asthma. https://www.cdc.gov/brfss/annual_data/2019/pdf/codebook19_llcp-v2-508.HTML

**TABLE:** Prevalence of chronic conditions* reported by adults aged 18–34 years, by selected characteristics — Behavioral Risk Factor Surveillance System, United States, 2019

Characteristic	No.	% (95% CI)							
		Any chronic condition*^†^	Chronic condition^§^						
			Obesity	Depression	HBP	High cholesterol^¶^	Asthma	Arthritis	Other
**Overall**	67,104	53.8 (53.1–54.5)	25.5 (24.9–26.1)	21.3 (20.8–21.8)	10.7 (10.3–11.1)	9.8 (9.3–10.2)	9.2 (8.9–9.6)	5.9 (5.6–6.2)	7.4 (7.1–7.8)
**Sociodemographic characteristics**									
**Sex**									
Men	35,131	50.0 (49.0–50.9)	23.2 (22.4–24.0)	15.8 (15.2–16.4)	13.4 (12.8–14.0)	10.0 (9.4–10.6)	7.1 (6.6–7.5)	4.9 (4.5–5.3)	6.5 (6.0–7.0)
Women	31,973	57.7 (56.7–58.7)	27.9 (27.0–28.8)	27.0 (26.2–27.9)	7.8 (7.4–8.3)	9.6 (8.9–10.3)	11.5 (11.0–12.1)	6.9 (6.5–7.4)	8.4 (7.9–9.0)
**Age group, yrs**									
18–24	24,411	48.7 (47.6–49.8)	19.4 (18.5–20.3)	22.0 (21.2–22.9)	7.9 (7.3–8.4)	7.2 (6.6–7.9)	10.3 (9.7–10.9)	3.5 (3.2–3.9)	5.5 (5.0–6.1)
25–34	42,693	57.3 (56.5–58.2)	29.8 (29.0–30.5)	20.8 (20.2–21.4)	12.7 (12.2–13.2)	11.6 (11.0–12.2)	8.5 (8.1–9.0)	7.5 (7.1–7.9)	8.7 (8.2–9.2)
**Race and ethnicity**									
White, NH	42,674	56.4 (55.7–57.2)	23.9 (23.3–24.6)	27.0 (26.3–27.7)	11.5 (11.0–12.0)	9.4 (8.9–9.9)	9.9 (9.4–10.3)	7.4 (7.0–7.8)	7.1 (6.7–7.5)
Black, NH	5,990	56.8 (54.6–58.9)	33.7 (31.5–36.0)	16.0 (14.6–17.6)	12.5 (11.3–13.8)	10.0 (8.7–11.6)	11.6 (10.5–12.8)	4.9 (4.2–5.8)	8.7 (7.6–10.0)
Hispanic	10,853	52.4 (50.7–54.2)	29.2 (27.6–30.7)	14.6 (13.6–15.8)	9.4 (8.5–10.3)	10.5 (9.4–11.6)	7.8 (7.0–8.8)	3.8 (3.2–4.6)	8.2 (7.2–9.3)
Other/Multiple race, NH	7,587	40.6 (38.5–42.7)	15.9 (14.6–17.4)	13.8 (12.6–15.1)	7.7 (6.8–8.7)	10.0 (8.8–11.4)	6.7 (5.8–7.7)	4.1 (3.4–5.0)	6.0 (5.1–7.2)
Poverty level**									
<100% FPL	12,090	57.2 (55.3–59.1)	29.1 (27.6–30.6)	23.7 (22.3–25.2)	11.8 (10.8–12.8)	10.3 (8.9–11.9)	10.9 (9.9–12.0)	6.4 (5.8–7.2)	9.7 (8.8–10.7)
≥100% to <200% FPL	16,144	56.3 (54.7–57.9)	27.5 (26.2–28.8)	23.0 (21.9–24.1)	11.0 (10.3–11.8)	9.7 (8.7–10.7)	9.7 (8.8–10.7)	6.6 (6.0–7.3)	8.6 (7.8–9.4)
≥200% FPL	38,870	51.5 (50.5–52.4)	23.3 (22.5–24.1)	19.7 (19.0–20.4)	10.1 (9.7–10.7)	9.6 (9.1–10.2)	8.4 (8.0–8.9)	5.3 (4.9–5.7)	6.1 (5.6–6.6)
**Employment status** ^††^									
Employed	46,781	53.7 (52.9–54.5)	26.1 (25.5–26.8)	19.4 (18.8–20.0)	11.0 (10.5–11.4)	9.7 (9.2–10.2)	8.3 (7.9–8.7)	5.6 (5.3–6.0)	7.0 (6.6–7.5)
Unemployed	4,449	62.3 (59.6–64.8)	29.2 (26.8–31.7)	30.9 (28.8–33.1)	13.5 (12.0–15.1)	11.4 (9.6–13.5)	12.5 (10.9–14.3)	7.9 (6.7–9.4)	10.1 (8.5–12.1)
Student	9,406	45.8 (44.1–47.5)	15.9 (14.6–17.3)	21.1 (19.8–22.5)	7.1 (6.3–8.0)	7.8 (6.9–8.8)	10.0 (9.1–11.0)	2.7 (2.2–3.2)	4.5 (3.8–5.2)
Other	5,857	62.6 (60.2–64.9)	35.1 (32.5–37.9)	28.4 (26.5–30.3)	12.8 (11.5–14.1)	12.7 (11.2–14.3)	12.7 (11.3–14.4)	11.9 (10.6–13.3)	13.6 (12.2–15.2)
**Education level** ^††^									
High school or less	24,690	55.6 (54.5–56.7)	28.5 (27.5–29.5)	20.9 (20.1–21.7)	11.9 (11.3–12.6)	9.4 (8.6–10.2)	9.4 (8.8–10.0)	6.0 (5.5–6.4)	9.0 (8.3–9.7)
Some college or more	42,196	52.4 (51.6–53.2)	23.2 (22.5–23.9)	21.7 (21.1–22.4)	9.8 (9.3–10.2)	10.0 (9.5–10.6)	9.1 (8.7–9.6)	5.8 (5.4–6.2)	6.2 (5.8–6.6)
**Disability** ^§§^									
Without disability	54,198	48.3 (47.6–49.1)	23.8 (23.2–24.4)	14.5 (14.0–15.0)	9.0 (8.7–9.4)	8.9 (8.4–9.5)	7.6 (7.2–8.0)	3.8 (3.6–4.1)	5.5 (5.1–5.8)
With disability	12,906	75.8 (74.3–77.1)	32.3 (30.9–33.7)	48.9 (47.4–50.4)	17.3 (16.3–18.4)	13.3 (12.3–14.3)	16.0 (15.0–17.0)	14.1 (13.2–15.1)	15.3 (14.3–16.4)
**Location characteristics**									
**Region** ^¶¶^									
Northeast	9,534	53.7 (52.2–55.3)	22.5 (21.3–23.8)	21.8 (20.6–23.1)	9.6 (8.7–10.6)	10.4 (9.5–11.5)	11.3 (10.4–12.3)	5.5 (4.8–6.2)	6.7 (5.9–7.5)
Midwest	19,093	55.7 (54.5–56.9)	27.3 (26.3–28.4)	23.8 (22.8–24.7)	10.6 (10.0–11.4)	8.8 (8.1–9.5)	10.2 (9.5–10.9)	6.9 (6.3–7.5)	6.9 (6.3–7.5)
South	20,422	55.6 (54.4–56.8)	28.0 (26.9–29.1)	21.2 (20.3–22.1)	11.5 (10.9–12.2)	10.4 (9.6–11.2)	7.9 (7.4–8.6)	6.3 (5.8–6.9)	8.7 (8.0–9.5)
West	18,055	49.3 (48.0–50.7)	21.8 (20.7–22.9)	19.2 (18.3–20.1)	10.0 (9.3–10.8)	9.3 (8.5–10.1)	9.3 (8.6–10.0)	4.6 (4.1–5.1)	6.3 (5.7–7.0)
Urbanicity***									
Urban	59,720	53.4 (52.7–54.1)	25.1 (24.5–25.7)	21.2 (20.7–21.8)	10.5 (10.1–10.9)	9.8 (9.3–10.3)	9.2 (8.8–9.6)	5.7 (5.4–6.0)	7.4 (7.0–7.8)
Rural	7,384	59.8 (57.6–62.1)	32.9 (30.7–35.2)	22.7 (21.1–24.5)	13.7 (12.3–15.2)	9.7 (8.1–11.5)	10.0 (8.8–11.3)	8.3 (7.3–9.5)	7.9 (6.9–9.1)
**Self-rated health status**									
**Fair or poor general health** ^†††^									
No	59,899	50.4 (49.7–51.2)	23.3 (22.7–23.9)	18.8 (18.3–19.3)	9.0 (8.7–9.4)	8.8 (8.3–9.3)	8.3 (7.9–8.7)	4.5 (4.2–4.8)	5.6 (5.3–6.0)
Yes	7,205	79.8 (77.9–81.5)	42.0 (40.1–44.0)	41.0 (39.1–42.9)	23.4 (21.9–25.1)	17.5 (16.0–19.2)	16.7 (15.4–18.1)	16.6 (15.3–18.1)	21.3 (19.7–23.1)
**Frequent physical distress** ^§§§^									
No	62,463	52.1 (51.4–52.8)	24.8 (24.2–25.4)	19.6 (19.1–20.1)	9.8 (9.5–10.2)	9.3 (8.8–9.7)	8.7 (8.3–9.0)	4.6 (4.4–4.9)	6.4 (6.1–6.8)
Yes	4,641	76.8 (74.6–78.9)	35.0 (32.6–37.5)	44.2 (41.8–46.7)	22.3 (20.3–24.3)	16.7 (14.7–18.8)	17.0 (15.4–18.8)	22.4 (20.4–24.6)	21.0 (18.9–23.3)
**Frequent mental distress** ^¶¶¶^									
No	54,922	48.8 (48.0–49.6)	24.4 (23.7–25.0)	14.1 (13.6–14.6)	9.4 (9.1–9.8)	9.2 (8.7–9.8)	8.0 (7.7–8.4)	4.7 (4.4–5.0)	6.2 (5.9–6.6)
Yes	12,182	76.1 (74.7–77.5)	30.5 (29.1–31.9)	53.7 (52.2–55.3)	16.3 (15.2–17.4)	12.4 (11.3–13.5)	14.8 (13.8–15.8)	11.1 (10.3–12.1)	12.7 (11.6–13.8)
**Health care coverage **									
**Access to health care** ^††^									
No	11,479	52.5 (50.9–54.1)	27.4 (26.0–28.9)	18.4 (17.3–19.6)	11.3 (10.3–12.2)	9.5 (8.5–10.6)	6.8 (6.1–7.6)	5.2 (4.7–5.9)	8.4 (7.5–9.4)
Yes	54,859	54.1 (53.4–54.9)	25.1 (24.4–25.7)	21.9 (21.3–22.5)	10.6 (10.2–11.0)	9.9 (9.4–10.4)	9.9 (9.5–10.3)	6.0 (5.7–6.3)	7.2 (6.8–7.6)

**Abbreviations:** FPL = federal poverty level; HBP = high blood pressure; NH = non-Hispanic.

* Behavioral Risk Factor Surveillance System respondents were classified as having an underlying chronic condition if they answered “yes” to having any of the following conditions (question number): depression (C06.09); HBP (C04.01); high cholesterol (C05.01); asthma (C06.04 and C06.05); arthritis (C07.01); other (C06.01, C06.02, C06.03, C06.06, C06.07, C06.08, C06.10, and C06.11). The questionnaire can be found at https://www.cdc.gov/brfss/questionnaires/pdf-ques/2019-BRFSS-Questionnaire-508.pdf. Obesity was defined as having a body mass index ≥30.0 kg/m^2^ based on self-reported height and weight.

^†^ Having any chronic condition was significantly (p<0.05) associated with all characteristics, except health care coverage.

^§^ Having obesity, depression, HBP, current asthma, arthritis, or other chronic conditions was significantly (p<0.05) associated with sex, age, race and ethnicity, poverty level, employment status, disability status, region, and all self-rated health characteristics; obesity, HBP, and arthritis were significantly (p<0.05) associated with urban-rural status; and obesity, HBP, and other conditions were significantly associated with education level. Obesity, depression, current asthma, arthritis, and other chronic conditions were significantly associated with health care coverage.

^¶^ Having high cholesterol was significantly (p<0.05) associated with age, employment status, disability status, region, and self-rated general, physical, and mental health.

** Poverty level is the ratio of total family income to FPL per family size (% FPL).

^††^ Sample size <67,104 because of missing data; multiple imputation has been used for all other characteristics and conditions.

^§§^ Adults were considered to have a disability if they reported having one or more of the following six disability types: hearing, vision, cognition, mobility, self-care, or independent living.

^¶¶^ https://www2.census.gov/geo/pdfs/maps-data/maps/reference/us_regdiv.pdf

*** Urban-rural status was categorized using the National Center for Health Statistics 2013 Urban-Rural Classification Scheme for Counties. https://www.cdc.gov/nchs/data/series/sr_02/sr02_166.pdf

^†††^ Fair or poor general health was defined based on responses to the question, “Would you say in general that your health is—excellent, very good, good, fair, or poor?”

^§§§^ Frequent physical distress was defined as responding ≥14 days to the question, “Now thinking about your physical health, which includes physical illness and injury, for how many days during the past 30 days was your physical health not good?”

^¶¶¶^ Frequent mental distress was defined as responding ≥14 days to the question, “Now thinking about your mental health, which includes stress, depression, and problems with emotions, for how many days during the past 30 days was your mental health not good?”

Consistent with having any condition, the prevalence of having obesity, depression, or high blood pressure was significantly associated with nearly all selected characteristics. Differences in the prevalence for having obesity were most noticeable between young adults aged 25–34 years (29.8%) and 18–24 years (19.4%), non-Hispanic Black persons (33.7%) and non-Hispanic White persons (23.9%), those who were unemployed (29.2%) or employed (26.1%) and a student (15.9%), those with (32.3%) and without (23.8%) a disability, and those living in rural (32.9%) and urban (25.1%) areas. Differences in the prevalence of having depression were most noticeable between females (27.0%) and males (15.8%), non-Hispanic White persons (27.0%) and non-Hispanic Black persons (16.0%) or Hispanic persons (14.6%), adults who were unemployed (30.9%) and employed (19.4%), and those with (48.9%) and without (14.5%) a disability. Differences in the prevalence of high blood pressure were most noticeable between males (13.4%) and females (7.8%), young adults aged 25–34 years (12.7%) and 18–24 years (7.9%), non-Hispanic Black persons (12.5%) and Hispanic persons (9.4%), those who were unemployed (13.5%) or employed (11.0%) and a student (7.1%), those with (17.3%) and without (9.0%) a disability, and those living in rural (13.7%) and urban (10.5%) areas. Prevalence of health-related risk behaviors was higher among those with any condition than among those without one (Figure 2).

**FIGURE 2:**
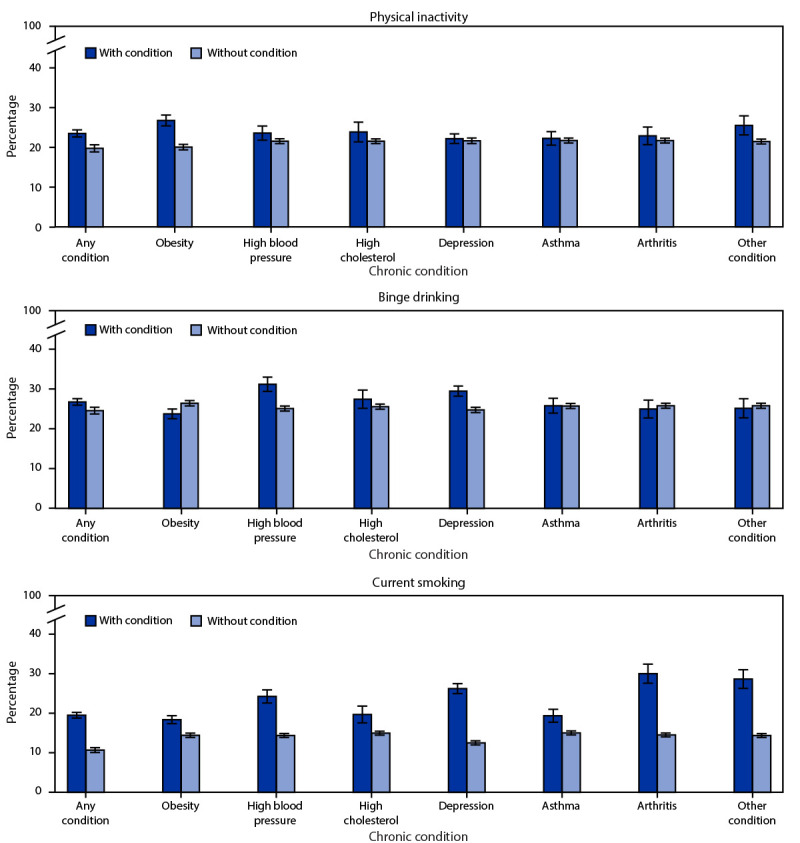
Percentage* of engaging in health-related risk behaviors,^†^ by adults aged 18–34 years with and without reported chronic conditions^§^ — Behavioral Risk Factor Surveillance System, United States, 2019 * 95% CIs indicated by error bars; prevalence of physical inactivity is significantly different (p<0.05) between those with and without the following conditions: any condition, obesity, high blood pressure, and other; prevalence of binge drinking is significantly different (p<0.05) between those with and without the following conditions: any condition, obesity, high blood pressure, and depression; prevalence of current smoking is significantly different (p<0.05) between those with and without each condition. ^†^ Health-related risk behaviors were defined as follows: physical inactivity (other than regular job, not engaging in any physical activities or exercises such as running, calisthenics, golf, gardening, or walking for exercise during the past month); binge drinking (males having five or more drinks on one occasion, females having four or more drinks on one occasion); current smoking (smoking ≥100 cigarettes in one’s lifetime and still smoking on at least some days). ^§^ Other includes the following conditions: chronic obstructive pulmonary disease, cancer, diabetes, heart disease/stroke, and kidney disease.

## Discussion

Approximately one half of young adults reported at least one chronic condition, with the most common being obesity (25.5%), depression (21.3%), and high blood pressure (10.7%). Young adults with any chronic condition were more likely than those without a chronic condition to report binge drinking, smoking, and physical inactivity. Because chronic conditions become more prevalent with age, a focus on prevention and risk factors is essential for health across the life span. These findings highlight the importance of increasing the availability of evidence-based strategies tailored to young adults to improve the prevention, treatment, and management of chronic conditions.

Research among the adult population has found differences in the prevalence of specific chronic conditions by sociodemographic characteristics. For example, the prevalence of obesity was higher among adults aged 25–44 years than among those aged 20–24 years (*2*). Obesity prevalence was also highest among adults with a physical activity limitation disability (*2*). The prevalence estimates for obesity and hypertension were also elevated among non-Hispanic Black persons, those unemployed but previously working, and adults not living in a metropolitan statistical area (*3*). Long-standing inequities^§§^ across many chronic conditions might be reduced by addressing social determinants of health and removing systemic and long-standing barriers to practicing healthy behaviors (e.g., poor living and working conditions and racial discrimination) (*1*,*4*). Moreover, consistent with what is known regarding risk factors for chronic conditions (*5*), young adults who reported binge drinking, smoking, and physical inactivity were more likely to have at least one chronic condition than those who did not report these behaviors, and some of the common chronic conditions in this age group (obesity, high blood pressure, and high cholesterol) are metabolic risk factors for other chronic conditions (e.g., diabetes or heart disease).^¶¶^ Addressing health behaviors and intermediate conditions among young adults can help improve long-term health and well-being over the life course (*1*).

Including a developmental perspective and incorporating mechanisms and channels that specifically resonate with young adults might help improve the effectiveness of strategies to reduce the prevalence of chronic conditions among this group. However, health interventions and programs to help guide individual-, clinical-, and community-level strategies to improve chronic conditions in this population are limited (*1*). The National Academies report on Investing in the Health and Well-Being of Young Adults provides a set of recommendations across domains to develop evidence-based practices for young adults for medical and behavioral health, including prevention (*1*). For example, within the health care domain, the report recommends building on evidence-based practices shown to be effective in adults of all ages and adolescents to 1) identify and determine the efficacy of practices that might be promising in young adults, 2) identify practices that once modified are likely to be effective, and 3) support research to develop practices in young adults in areas identified as unlikely to be addressed with current practices (*1*). Within the public health infrastructure domain, the report recommends research 1) in the effectiveness of multilevel strategies in improving health outcomes and reaching hard-to-reach young adults, 2) on how social media influences health outcomes, and 3) to improve understanding of how social determinants of health and other factors contribute to health disparities among young adults (*1*). These recommendations provide a broad framework that can guide the development of effective strategies to improve the health of young adults.

The findings in this report are subject to at least two limitations. First, BRFSS data are self-reported and subject to recall and social-desirability biases. For example, prevalence of self-reported, physician-diagnosed chronic conditions might be underestimated; however, state-level prevalence of some conditions is consistent with estimates derived from electronic health records (*6*). Second, the median response rate of 49.4% might reduce generalizability; however, BRFSS uses a sophisticated weighting method (iterative proportional fitting) that does not require demographic information for small geographic areas, thereby reducing the potential for certain biases (*7*).

Approximately one half of young adults reported at least one chronic condition. Continued efforts are needed to help identify, develop, and modify, where necessary, effective strategies to prevent, treat, and manage chronic conditions in young adults. Public health professionals might consider tailoring individual- and community-level strategies to young adults.

**Summary**

**What is already known about this topic?**

Chronic conditions are common, costly, and major causes of death and disability. Addressing conditions in young adulthood can help slow disease progression and improve well-being across the life span; however, recent estimates among young adults have not been reported.

**What is added by this report?**

In 2019, 53.8% of adults aged 18─34 years had at least one chronic condition, and 22.3% had more than one condition. Prevalence of any as well as specific chronic conditions varied by population subgroup.

**What are the implications for public health practice?**

Coordinated efforts might help improve the availability of evidence-based interventions, policies, and programs that are effective in preventing, treating, and managing chronic conditions in young adults.

## Footnotes

*
https://www.cdc.gov/chronicdisease/index.htm

†
https://www.cdc.gov/brfss/about/index.htm

§
https://www.cdc.gov/brfss/annual_data/2019/pdf/2019-sdqr-508.pdf

¶ Binge drinking was defined as males having five or more drinks on one occasion and females having four or more drinks on one occasion. Smoking was defined by self-report of smoking >100 cigarettes in one’s lifetime and still smoking some days or every day at the time of the survey. Physical inactivity was defined as responding “no” to the question, “During the past month, other than your regular job, did you participate in any physical activities or exercises such as running, calisthenics, golf, gardening, or walking for exercise?”

** Overall, 3% of the data were missing; variables with the most frequent missing data were poverty level (20%), high cholesterol (19%), and obesity (10%). The SAS multiple imputation procedure using the fully conditional specification method generated multiple (five) data sets (https://support.sas.com/resources/papers/proceedings15/2081-2015.pdf). As part of the SUDAAN procedure, the estimates reported were obtained by combining the results from all the imputed data sets.

†† 45 C.F.R. part 46, 21 C.F.R. part 56; 42 U.S.C. Sect. 241(d); 5 U.S.C. Sect. 552a; 44 U.S.C. Sect. 3501 et seq.

§§
https://www.cdc.gov/socialdeterminants/index.htm

¶¶
https://www.who.int/news-room/fact-sheets/detail/noncommunicable-diseases
